# Tuberculous Pachymeningitis Presenting as a Diffused Dural Thickening in a Patient with Chronic Headache and Recurrent Neurological Abnormalities for More than a Decade: A Case Report and a Review of the Literature

**DOI:** 10.1155/2018/3012034

**Published:** 2018-10-01

**Authors:** C. L. Fonseka, T. E. Kanakkahewa, S. D. A. L. Singhapura, J. S. Hewavithana, L. P. Kolambage, H. M. M. Herath, K. D. Pathirana, Thilak Priyantha Weeraratna

**Affiliations:** ^1^Consultant Physician, Department of Internal Medicine, Faculty of Medicine, University of Ruhuna, Galle, Sri Lanka; ^2^Consultant Physician, University Medical Unit, Teaching Hospital Karapitiya, Galle, Sri Lanka; ^3^Consultant Radiologist, Department of Radiology, Teaching Hospital Karapitiya, Galle, Sri Lanka; ^4^Consultant Neurologist, Department of Internal Medicine, Faculty of Medicine, University of Ruhuna, Galle, Sri Lanka

## Abstract

**Background:**

Tuberculous pachymeningitis is a rare form of extrapulmonary tuberculosis usually suspected from the detection of thickening of the dura in contrast-enhanced magnetic resonance imaging. Progressive nature of the disease can lead to chronic headache with focal neurological signs due to compression from the thickened dura.

**Case Report:**

We report a 40-year-old female who presented with chronic headache over a decade associated with recurrent neurological abnormalities including optic neuritis, hemisensory loss, migraine, facial nerve palsy, and recurrent vertigo. Although there was an initial perceived response to steroids, the patient had a subsequent progressive course. On investigations, she was found to have a diffused dural thickening on contrast MRI with a strongly positive Mantoux test with caseating necrotizing granulomatous inflammation on dural histology. With initiation of antituberculous medication with steroids, the patient markedly improved, and the medication for tuberculosis was continued for a year with good response.

**Conclusion:**

Primary tuberculous pachymeningitis should be suspected in a patient complaining of prolonged headache with focal neurological signs when MRI evidence of dural thickening is detected, and another focus of tuberculosis was not found. Prompt suspicion with image-guided dural biopsy for histology would help to confirm the diagnosis.

## 1. Background

Tuberculous meningitis (TBM) is a serious and life-threatening presentation of extrapulmonary tuberculosis. Delay in the diagnosis and treatment of this condition can result in significant morbidity and mortality [1]. Even in tuberculosis- (TB-) prevalent countries, the diagnosis of TBM is difficult at times and requires a high degree of clinical suspicion. Direct cerebrospinal fluid (CSF) examination & polymerase chain reaction of CSF (TB-PCR) are specific diagnostic tools but have varied sensitivities, and mycobacterial culture is time-consuming and can delay timely diagnosis [1]. MRI or CT brain may show localized basilar meningeal enhancement, with or without hydrocephalus.

Other than TBM with leptomeningeal involvement, CNS tuberculosis can present in several ways such as intracranial tuberculomas, tuberculous pachymeningitis, and spinal tuberculous arachnoiditis. TB meningitis and intraparenchymal granulomatous lesions are the common presentations of CNS tuberculosis [2, 3]. Cranial pachymeningitis due to involvement of the dura mater by tubercular infection is quite rare. Pachymeningitis has been described as diffuse or localized thickening of the cranial dura mater with or without associated inflammation [2, 3].

Regarding cranial pachymeningitis due to any aetiology, clinical manifestations vary depending on the location of the pathology, and the affected patients commonly present with manifestations of increased intracranial pressure, such as headache, or progressive neurologic deficits arising due to compression of neural structures by the thickened dura mater such as cranial neuropathies including vestibular involvement and seizures [4]. While a number of causes of dura mater thickening have been reported, including trauma, infection, autoimmune diseases, connective tissue diseases, sarcoidosis, and malignancy, many cases of cranial pachymeningitis were thought to be idiopathic [4]. However, the finding that some idiopathic cases show a beneficial response to antituberculous therapy suggests that a proportion of them may be caused by tuberculosis and it could be missed [5]. Although pachymeningeal changes are commonly found over the cerebral convexities and in basal or tentorial locations [2, 6], falcine involvement has been also described [3]. Radiologic findings include diffuse localized thickening of the dura mater, with homogenous enhancement following contrast administration especially in T1-weighted images of MRI. There is often an underlying white mater oedema and sulcal effacement, which presumably indicates leptomeningeal involvement [2, 3]. However, patients may not have any imaging feature unique to tuberculous pachymeningitis, and also radiologic findings may be misleading, suggesting other diagnoses such as meningioma or meningioma en plaque [7–9]. CSF analysis may reveal a mildly elevated protein concentration, a pleocytosis with mainly lymphocytes or no abnormality at all [3].

Biopsy of the thickened dura revealing necrotizing granulomatous inflammation with caseation with Langerhans giant cells will help to arrive at a definitive diagnosis in dural TB. PCR for *Mycobacterium tuberculosis* DNA on CSF has been reported positive infrequently in localized cranial pachymeningitis denoting that it cannot be relied upon to exclude diagnosis of TB. In addition, a negative Mantoux test cannot be relied upon to exclude the diagnosis, as the Mantoux test has been negative in several biopsy-proven cases reported in the literature [9, 10]. Although, CSF TB-PCR may reveal negative results, there were many instances where tissue PCR of the dural biopsy specimen became positive even when acid-fast bacilli were not detected microscopically [3]. In some cases, the diagnosis has been presumed solely on the basis of a beneficial response to antituberculous therapy. Indeed, given the often-progressive nature and relatively poor prognosis of the idiopathic form, a trial of treatment with antituberculous therapy and steroids may even be considered in cases where tuberculosis is suspected but not proven [2]. Therefore, the diagnosis should be presumed on the histology findings and Mantoux testing where presence of caseation will suggest tuberculosis in a tuberculosis-prevalent setting. As the TPM numbers are in small numbers, data on sensitivity, specificity, and predictive values of testing dural specimens were not available.

We report a case of tuberculous pachymeningitis that may have been continuing for almost a decade in a prevalent setting. This unique case describes the dilemmas in diagnosis and management issues faced.

## 2. Case Presentation

A 40-year-old female presented with chronic headache with infrequent exacerbations. She presents with worsening headache for three months with associated vertigo, nausea, and vomiting not responding to analgesics or vestibular sedatives. Her symptoms initially started twelve years ago as a diffused mild headache, which persisted through the day. Gradually, the headache worsened to a severe headache episodically associated with vertigo, nausea, and vomiting. These episodes lasted for two to three days and got resolved. She was treated with flunarizine for suspected basilar migraine but did not show any response. From the last year, she had monthly exacerbations of headache associated with distressing vertigo, unsteadiness of gait, and right-sided body numbness. In between these episodes, she had a significant dull diffuse headache not responding to simple analgesia. She did not complain of fever or night sweats but had constitutional symptoms lasting for several months. All of these symptoms severely affected her daily activities and functionality.

During the last 17 years, she had repeated episodes of neurological deficits. Even before the headache appeared, she has presented with visual impairment of the right eye and right lateral rectus palsy and was treated as retrobulbar neuritis with good response to methylprednisolone. One year later, she developed left-sided visual impairment, which fully responded to methylprednisolone. MRI imaging at that time revealed normal results. Few months after this event, she got admitted with right hemisensory loss with hemiplegia, and a demyelination disease or hemiplegic migraine was suspected. Second MRI was performed at this admission, and no abnormalities were detected again. Eight years ago, she had developed a left lower motor type facial nerve palsy, which was attributed to Bell's palsy. Within the last year, she was diagnosed to have depression and anxiety for which she was treated for few months. Other than the first two instances, she was not treated with steroids thereafter. She did not complain of weight loss and did not have constitutional symptoms or chest symptoms during these periods.

On examination, she is an averagely built female with a BMI of 23 kg/m^2^. She is afebrile, pale, and did not have lymphadenopathy. Her GCS was 15/15, and she was conscious and rational with normal pupillary response, visual acuity, visual field examination, and fundoscopy. There was no neck rigidity, and she had residual left lower motor VII palsy. She had an ataxic broad-based gait with unsteadiness. Upper and lower limb examination is clinically normal. Her respiratory, cardiovascular, and abdomen examinations were unremarkable.

**Figure 1:**
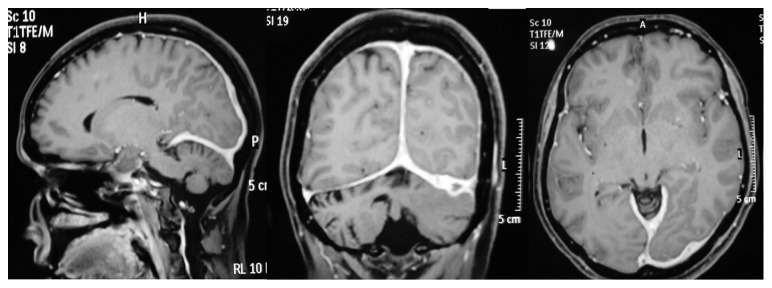
Sagittal, coronal, and transverse sections showing diffused dural thickening with contrast enhancement.

**Figure 2:**
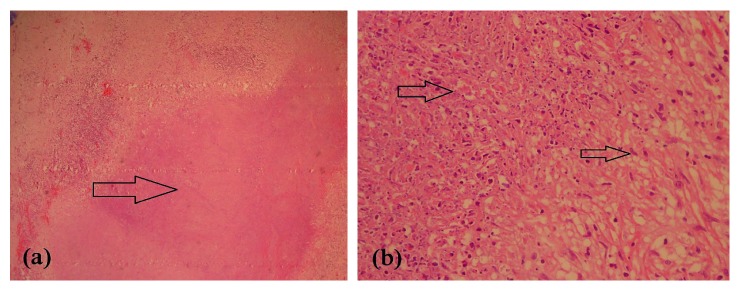
(a) Large areas with caseous necrosis (H&E × 40). (b) Arrow on the left—areas of caseous necrosis; eosinophilic, granular, and amorphous material with scattered nuclear debri (H&E × 400); arrow on the right—epithelioid histiocytes lining the areas with caseous necrosis (H&E × 400).

**Figure 3:**
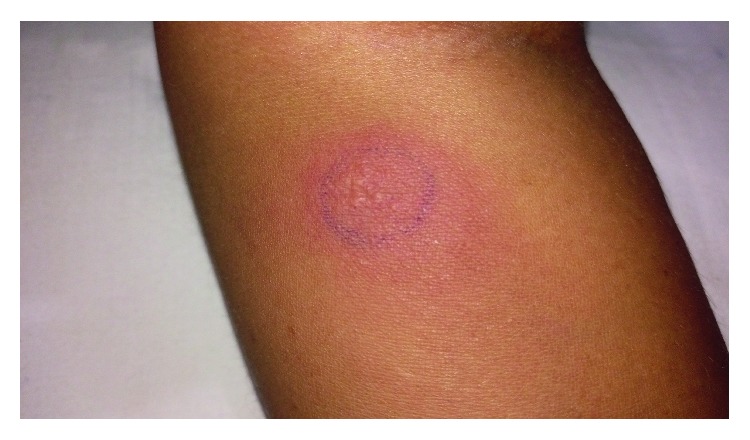
Positive Mantoux test of 25 mm.

Investigations revealed a hemoglobin count of 9 g/dL with normal white cells and platelets. ESR was elevated to 86 mm/1^st^ hour. Renal- and liver-related biochemical investigations were normal with an alkaline phosphatase within the normal range. Initial MRI scans of the brain done 10 years ago did not reveal any abnormalities such as demyelination, optic nerve enhancement of focal lesions in the cerebrum, or cerebellum. CSF examination revealed an elevated protein level of 55 mg/dl with normal glucose and cells with negative oligoclonal bands or TB-PCR. Vasculitis investigations including ANA, ANCA (ELISA and Immunofluorescence), and RF were negative. Syphilis serology and HIV testing were also negative. Chest radiograph, ultrasound abdomen, and CT scan of chest and abdomen did not reveal any mediastinal lymphadenopathy or focal lesions in visceral organ or evidence of any malignancy. Serum ACE levels (19 *µ*/l) and ionized calcium levels were normal. We performed a new MRI scan of the brain with contrast, which revealed a diffuse and patchy meningeal thickening and enhancement mainly in the right frontoparietal and left occipital regions with a minor enhancement of bilateral optic sheaths (Figure 1). Her NMO antibodies were normal, and the MRI did not show any areas of demyelination. Therefore, she underwent a dural biopsy from the thickened dura, which revealed large areas of caseous necrosis surrounded by epithelioid histiocytes and lymphoid cells with a few isolated giant cells in the adjacent vicinity (Figure 2). There were no features of vasculitis or sarcoidosis. TB-PCR of tissue and acid-fast bacilli were negative. Final conclusion was necrotizing granulomatous inflammation suggestive of dural tuberculosis. This diagnosis was presumed by the presence of necrotizing granulomatous necrosis with caseation with a strongly positive Mantoux test of 25 mm (Figure 3), and later was supported by a marked response to antituberculous medication.

We initiated her on antituberculous therapy (all four drugs for 3 months and 9 months of rifampicin and isoniazid) without streptomycin as she is already having vestibular symptoms. Steroids were added concurrently (1 mg/kg) and was continued for 6 weeks and was tailed off over a month. She experienced a marked improvement of her headache, and she could do her daily activities normally. After a year of anti-TB medication and follow-up, she did not complain of any worsening of symptoms.

## 3. Discussion

Tuberculous pachymeningitis (TPM) is an uncommon presentation of CNS TB. Unlike other varieties of CNS TB, the symptoms could progress for many months or years described as 5 months to 4 years in a certain cohort [2]. In our patient, it has continued over a decade and may have been misinterpreted as optic neuritis, migraine, stroke, Bells' palsy, and depression. In the initial episodes where optic neuritis was suspected, she had shown a response to steroids as well. It seems that the patient was fortunate that a dissemination of TB has not occurred in instances. Other explanation would be whether the patient had a prior optic neuritis where she responded to steroid and due to the immunosuppressed state, she may have got tuberculosis of the dura mater being in a TB-prevalent country. When we assessed the initial MRI we found that it was normal. Therefore, we could speculate that the dural thickening noted in the MRI may have occured due to chronic inflammtion over many years.

From the reported cases of TPM, there have been quite disabling and fatal manifestations. Most patients with TPM had headache as the most common symptom. Other symptoms included vomiting, impaired vision, diplopia, and cranial nerve palsies. None of these patients complained of fever [2]. While investigations were carried out to find the aetiology for a suspected TPM, it would be imperative to find out for a pulmonary focus of TB. In a series of seven patients with TPM, four had concurrent pulmonary TB signifying the importance of this fact. Most (86%) of the cases in that series also had focal thickening of dura with en plaque appearance that may mimick a meningioma. Lesser number of patients may have diffused dural thickening. A good clinical response in five of their patients who were treated with antituberculosis drugs alone also confirms that surgery is not always necessary, specially when isolated cranial TPM is present [2].

Craniocervical TPM is rarely reported in the literature and has been shown to present with associated hemiparesis or para/quadriparesis. Yamashita et al. described a patient with suspected hypertrophic pachymeningitis in the posterior fossa and cervical spine with a negative Mantoux test, who presented with progressive vertigo tinnitus; numbness of body; and dysphagia with gait disturbances with left-sided VII, VIII, IX, and XII cranial nerve involvement, who subsequently developed respiratory arrest and quadriparesis. This patient had caseous necrosis and acid-fast bacilli in dural histology. The patient succumbed despite decompressive craniectomy with excision of dura and antituberculous therapy, which may have been due to the delay in starting treatment [11]. Another patient with craniocervical TPM presented with history of progressive quadriparesis and stiffness of neck for 2 years and dysphagia to liquid for past 3 months. Her condition rapidly deteriorated when corticosteroids were prescribed. As suggested by the elevated CSF proteins with lymphocytosis and a positive Mantoux test, she was treated with antituberculous medication and improved later [12].

While investigating for probable clues to suggest TB, it has been evident that in some patients, the Mantoux test and CSF TB-PCR can become negative. But it would be important to biopsy the dura and look for necrotizing or caseating granulomas in dura and test tissue for TB-PCR. Thurtell et al. described two cases and Khawcharoenporn et al. described a case where tissue histology suggested TB and tissue PCR for TB also became positive [3, 13]. However, the sensitivity would depend on selecting the most appropriate site for dura guided by imaging for tissue histology. These patients showed good response to anti-TB medication, while the latter was given concurrent corticosteroids.

Dural-based tuberculomas may have different appearance on MRI unlike the homogenous enhancing thickening which is observed. For example, a patient had multiple dural-based tuberculomas rather than the usual homogenous enhancing dura with en plaque appearance in pachymeningeal tuberculosis with dural thickening [14]. Another young male had features of increased intracranial pressure, and there was an enhancing dural-based lesion in the left frontoparietal region. In view of herniation syndrome, urgent surgical decompression of the lesion was performed, and the histopathology showed features of tuberculosis [15]. Other than the above cases, intracranial tuberculoma of the dura mater with or without bone destruction has been reported [16, 17]. Also, dural-based soft tissue nodular mass with diffuse homogenous enhancement with prominent vasogenic oedema involving the underlying cerebral hemisphere was also reported [18]. One child was reported to have TPM associated with spinal tuberculomas presenting as progressive hemiparesis [19].

The duration of antituberculous medication for TPM is uncertain. It has been demonstrated that in tuberculomas, even after 18 months of antituberculous therapy, more than two-thirds have partial histological resolution. This suggests that more prolonged duration of therapy guided by resolution on imaging is needed [20]. A prolonged duration of antituberculous medicaition decided on an individual basis would be necessary in TPM as the antibiotic penetration to the dura could be theoretically low due to dural thickening.

Other differential diagnoses can closely resemble the current presentation of neurosarcoidosis. But, the presence of highly positive Mantoux is expected to be negative in neurosarcoidosis. Also, low CSF glucose and lack of hilar lymphadenopathy argue against the diagnosis of sarcoidosis.

Idiopathic hypertrophic pachymeningitis (IHP) was diagnosed and reported in many patients in the literature when an etiological factor is not found. In this condition, bilateral visual loss with headache and MRI evidence of frontal dural thickening with optic nerve encasement has been reported [5]. Some reports with IHP showed extensive cranial nerve involvement in CN II, VI, VII, X, and XII in different combinations and visual impairment with either optic atrophy with observed response to steroids [7]. CNS parenchymal features such as memory loss, hemiparesis, and wide-based gait with ataxia were uncommon. Our patient had suspected optic neuritis and unilateral facial nerve palsy that would have been due to a similar mechanism, and parenchymal features are also theoretical possibilities, especially when underlying vasogenic oedema is present with TPM.

## 4. Conclusions

Hypertrophic pachymeningitis is a rare form of tuberculosis that needs to be considered in differential diagnoses in patients with prolonged headache with focal neurological signs. Enhancing dural thickening in MRI would point towards the diagnosis, and image-guided dural biopsy with histology would be helpful in confirming the diagnosis.
